# Editorial: NMDA receptors in physiology and disease

**DOI:** 10.3389/fnsyn.2023.1163459

**Published:** 2023-03-02

**Authors:** María Verónica Baez, Julien P. Dupuis, Gaston Diego Calfa

**Affiliations:** ^1^Instituto de Biología Celular y Neurociencia (IBCN)-CONICET, Buenos Aires, Argentina; ^2^1UA de Histología, Embriología, Biología Celular y Genética, Facultad de Medicina—Universidad de Buenos Aires (UBA), Buenos Aires, Argentina; ^3^Université de Bordeaux, CNRS, Interdisciplinary Institute for Neuroscience (IINS), UMR 5297, Bordeaux, France; ^4^Instituto de Farmacología Experimental Córdoba (IFEC)-CONICET, Departamento de Farmacología, Facultad de Ciencias Químicas, Universidad Nacional de Córdoba, Córdoba, Argentina

**Keywords:** postsynaptic receptors, synaptic plasticity, learning and memory, development, disease

## Introduction

N-methyl D-aspartate glutamate receptors (NMDAR) play essential roles in the central nervous system, ranging from synaptogenesis to experience-dependent adaptation and higher cognitive functions. Not surprisingly, impairments in NMDAR signaling have been associated with a broad range of devastating neurological and psychiatric conditions including schizophrenia, bipolar disorder, depression, Alzheimer's, Huntington's, and Parkinson's diseases. Thus, understanding how the biogenesis, molecular architecture, surface trafficking, subcellular localization, gating properties, synaptic anchoring and organization of NMDAR shape physiological brain functions, and dissecting how hypo- or hyperfunctions of NMDAR contribute to the etiology of brain conditions has attracted growing attention and efforts. Over the past two decades, the developments of super-resolution microscopy, cryo-electron microscopy, chemogenetics, optogenetics, and optopharmacology have allowed breakthrough discoveries on the physiology of NMDAR and have improved significantly our understanding of their contributions to brain functions in physiological and diseased states. In this Research Topic, we have collected original and review articles highlighting recent progress in dissecting the mechanisms of NMDAR expression, synaptic function, and contribution to circuit dysfunctions in pathological conditions.

## Papers in this Research Topic

### Quality control of NMDAR folding and expression

Quality control mechanisms are essential processes ensuring that proteins are correctly processed and addressed in order to perform their physiological functions and maintain cellular homeostasis. Benske et al. provide a comprehensive review of past and recent literature on the cellular mechanisms ensuring that only correctly folded and assembled NMDAR exit the endoplasmic reticulum (ER) and reach the plasma membrane. They recapitulate the successive steps and related quality control processes supporting the biogenesis of GluN1, GluN2A, and GluN2B subunit-containing NMDAR, from (i) folding, assembly into tetramers and post-translational modifications in the ER, (ii) forward trafficking to the Golgi apparatus *via* canonical and non-canonical pathways, to (iii) vesicular export and delivery to the plasma membrane. In parallel, they detail the clearance pathways allowing elimination of misfolded proteins and maintenance of NMDAR homoeostasis. The authors also compile examples of disease-associated NMDAR variants exhibiting gain-of-function or loss-of-function deficits, urge the need of further exploring the consequences of these variants on receptor biogenesis, and discuss the therapeutic potential of approaches based on restoring a proper folding, assembly, surface trafficking or degradation of NMDAR in these conditions.

### Synaptic contributions of NMDAR at resting membrane potential

AMPAR respond rapidly to the brief increases in glutamate concentration in the synaptic cleft following vesicular release and support most of rapid neurotransmissions at excitatory synapses. In “*Synaptic NMDA receptor activity at resting membrane potentials*,” Chiu and Carter show that NMDAR may also contribute significantly to synaptic events at resting membrane potential in physiological ionic conditions, both at cortical L4-L2/L3 synapses and in hippocampal CA1 neurons. The authors observed NMDAR currents at all voltages essayed even in the absence of AMPAR-mediated depolarization. These are controversial results as NMDAR are believed to act as molecular coincidence detectors, only activated upon concomitant glutamate release and membrane depolarization caused by AMPAR activation. They raise challenging questions on the contribution of NMDAR to the functional integration of synaptic signals and call for further investigations.

### MK801-elicited behavioral deficits as a model to study schizophrenia

MK-801, a non-competitive NMDAR antagonist, is often used as a pharmacological model to elicit behavioral deficits in rodents mimicking some of the symptoms associated with schizophrenia. The review by Xu et al. summarizes recent literature on MK-801 induced cognitive impairments in rodents, discusses its relevance to schizophrenia research, and advertises how and through which mechanisms exposure to an enriched environment at different stages of the life of the animals ameliorates cognitive impairments caused by MK-801-elicited NMDAR hypofunction. The authors provide valuable information regarding the use of MK-801 as an experimental model to explore the cognitive deficits associated with schizophrenia in rodents and convincingly advocate for more efforts in understanding of how environmental enrichment intervention could contribute, at least from a preventive point of view, to alleviate these deficits.

Using this paradigm, Huang et al. dissect the circuits involved in MK-801-triggered behavioral deficits. The authors report that the anterior cingulate cortex (ACC) and basolateral amygdala (BLA) are involved in the social and cognitive impairments induced by MK-801 treatment. They show that glutamatergic projections from the BLA to ACC are active during social interactions and cognitive tests, and that chemogenetic and optogenetic manipulation of this neural circuit alleviates the negative behavioral manifestations induced by MK-801. Overall, these results improve our comprehension of the core mechanisms responsible for behavioral impairments in animal models of schizophrenia and open new perspectives on potential targets for therapeutic intervention to treat schizophrenia-related symptoms.

## Author contributions

MB, JD, and GC wrote the manuscript. All authors contributed to the article and approved the submitted version.

